# Beyond categorisation: refining the relationship between subjects and objects in health research regulation

**DOI:** 10.1080/17579961.2021.1898314

**Published:** 2021-03-18

**Authors:** Catriona McMillan, Edward Dove, Graeme Laurie, Emily Postan, Nayha Sethi, Annie Sorbie

**Affiliations:** School of Law, University of Edinburgh, Edinburgh, UK

**Keywords:** Subject, object, health research, liminality, health data

## Abstract

In this article, we argue that the relationship between ‘subject’ and ‘object’ is poorly understood in health research regulation (HRR), and that it is a fallacy to suppose that they can operate in separate, fixed silos. By seeking to perpetuate this fallacy, HRR risks, among other things, objectifying persons by paying insufficient attention to human subjectivity, and the experiences and interests related to being involved in research. We deploy the anthropological concept of liminality – concerned with processes of transformation and change over time – to emphasise the enduring connectedness between subject and object in these contexts. By these means, we posit that regulatory frameworks based on *processual regulation* can better recognise and encompass the fluidity and significance of these relationships, and so ground more securely the moral legitimacy and social licence for human health research.

## 1. Introduction

It is a near-universal legal truism that almost all regulated entities are held to fall into one of two categories: subject or object (classically: ‘person’ or ‘thing’). Within these two broad legal realms, but particularly with respect to ‘objects’, further ontological and moral demarcation usually occurs, conferring differing degrees of legal protection in accordance with the relative value that society places on the object in question. For example, we place different social, monetary and moral value on different ‘objects’, from pet animals, to historical artefacts, to ideas. It is no different within the field of health research regulation (HRR). The objects of HRR are myriad; they include ‘personal data’, DNA and RNA, cell lines, ‘human tissue’, ‘gametes’, and a range of legally prescribed embryos’, and all are bounded in law by their own definitions, frameworks, and rules of production, storage and use in health research.^1^ As a technocratic domain that is fundamentally concerned with managing risks to humans, HRR regimes are understandably focussed on the definition and categorisation of suitable objects of regulatory capture – such as what counts as ‘personal data’^2^ or which kinds of embryo can be created for research purposes^3^ – but there is often a deep irony that also arises, viz., the focus is then lost from the person(s) to whom these objects relate or from whom they have been derived, or who have contributed materially to the creation of these objects.^4^

From the annals of medico-legal lore, we have the infamous *Moore* case in which it was held that the cell line derived from Mr Moore’s spleen was ‘factually and legally distinct’ and on this basis, his claim to some legal interest in the cell line was refused by the Supreme Court of California;^5^ equally, in the *Source Informatics* case in the UK, it was held to be no breach of confidence when identifiers were stripped from patient data to allow those data to be passed on to third parties for statistical analysis purposes, despite the lack of consent to this from the subjects concerned.^6^ Indeed, the role of such anonymisation techniques in ‘breaking’ the connection between subject and object also finds considerable support in many statutory regimes of HRR, such as using personal data or human tissue for research purposes.^7^ In stark contrast, extensive social science literature shows that citizens continue to experience connection with the objects that they donate to health research or which are produced from their data or tissue to promote new scientific research, even if they are anonymised.^8^ A crucial question therefore arises: in legally fixing these ‘objects’ in pre-determined categories, does current HRR sufficiently capture the subjective, experiential dimensions of health research processes and the persons necessarily involved?

To better understand the nature of the relationship between human research participants (conceived of as subjects in more than one sense) and objects within the research process (a process that we suggest is fluid rather than fixed), we employ the concept of liminality: this is the anthropological concept coined in the early twentieth century by Arnold van Gennep: ‘ … [d]eveloped to make sense of ritual, structure, and agency, the notion of liminality refers to a threshold phase characterised by uncertainty, possibility, marginality, and transformation.’^9^ Given that health research is precisely concerned with uncertainty and transforming materials to produce new human understandings, this conceptual lens helps to reveal – as we will argue – that the relationship between the legal categories of ‘subject’ and ‘object’ is poorly understood, and that existing categorisations within HRR are inflexible and insufficient by obscuring the important transformations that take place between subjects and objects.

As we have shown elsewhere,^10^ liminality focuses attention on process and transition as experienced by human beings at times of change in their lives. In particular, the important change that occurs is to the *status* of persons,^11^ and the classic transitions are those from childhood to adulthood, from singledom to state/religious-sanctioned union, from wellness to illness, and from illness to death. The processes in question – when designed correctly – ought to support those persons and lead them through and out of the liminal phase. It is important to do so because liminality can represent the breakdown of order and pre-existing norms and social structures; liminality, therefore, is inherently uncertain, and can be chaotic and disruptive if not recognised and managed well. For present purposes, we posit that the processes of conducting human health research can usefully be subjected to the lens of liminality. This is so for two reasons: first, the nature of research itself is inherently transformative and uncertain, as suggested above.^12^ Second, the act of taking part in research is neither morally nor socially neutral. While this is self-evident in the case of clinical trials participation, we suggest that even the act of donating tissue or giving permission to use personal data in research involves a change of social status for the persons involved: they are transformed from everyday citizens into research participants.^13^ As we propose in Part 3 of this article, it may also transform them in broader and more fundamental ways. A liminal framing further suggests that we must follow all of this process through because the research endeavour is, in fact, comprised of a series of thresholds and phases, each with different implications and actors (such as recruitment of participants, and/or legitimate obtaining and use of data and tissues; involvement of research nurses and clinicians, and compliance with a host of regulatory approvals). Manifestly, ethical and socially responsible research does not begin and end with a successful ethical review. The social value of the research must also be realised (or at least there must be a reasonable prospect of this). Seen in this light, the entire research endeavour is inherently a liminal experience from the perspective of the individual *who becomes a research participant*. We are transformed by this process of being involved in research. This change of status in itself need not be great, nor necessarily significant to the lives of particular individuals. Nonetheless, we are confronted by the fact that it is a reality and a product of these biomedical practices. This, then, leads us to ask: *what can and should be done to lead people through and out of the liminality of human health research?*

It is our position that by binding objects of health research in their various regulatory silos, the law does not adequately capture the relationships between human research participants and the regulatory objects deriving *from them*, nor does it pay adequate attention to the experience of participants who may at times be *both* subject and object. In other words, by binding ‘objects’ within these silos, HRR does not capture persons’ (or subjects’) potential on-going interest(s) in objects relating to them and used in health research; moreover, these are interests that can persist even once any physical connection or de facto control has ceased.

This article proceeds as follows. In Part 2, we argue that while the law requires categorisation to provide a degree of certainty, doing so in HRR can deflect us from attending appropriately to the important subjective and experiential aspects of research. Part 3 explores three case studies that illustrate the most common subject-object transitions experienced in health research: (1) from object to subject (viz. research participation); (2) from subject to object (viz. data use); and (3) ‘subject/objects’, where something does not fit easily within this legal binary (viz. embryos *in vitro*). Finally, Part 4 draws from these case studies to offer a new framework – grounded in the idea of *processual regulation*^14^ – that provides a more nuanced way of capturing these three transitions. We argue that by employing processual regulation, it is possible to better reveal the subjective, experiential and fluid nature of the relation between subjects and objects in HRR and so ground more robustly the legitimacy of research.

## 2. Object categorisation in law and regulation: risks and realities

A core function of law and regulation is to prescribe and proscribe certain behaviours among human actors to achieve certain desired outcomes. A common thread that runs through this core function, no matter the law or regulation in question, is the steering of human behaviour through the mechanism of *object categorisation*. That is to say, law and regulation affect human behaviour by connecting desired conduct to an underlying object of regulatory attention, and this object (or set of objects) will often fall within one or more larger overarching legal categories. To categorise and ‘object-ify’ is the *modus operandi* of law. How things are sorted and grouped carries significant weight for the ways in which the force of law may be felt. As Sarat and colleagues put it:

In its basic operation, law attempts to create, police, and occasionally transgress social, spatial and temporal boundaries. […] Within law’s spatio-temporal grid, complex classifications are established, creating boundaries that define individuals, communities, acts and norms … .^15^

This is exemplified in much of the civil law legal system, where civil codes promulgated by legislatures lay out a corpus of general principles organised in a systematic way, such as the Napoleonic Code’s tripartite organisation into the law of persons, property law and commercial law. But it is equally reflected in the Common Law tradition, where statutes, regulations and judge-made law alike shape actions and identities of individuals and groups by creating boundaries around objects (broadly defined), be they personal data, embryos, organs, medical devices, investigational medicinal products, and so on. For example, the function of data protection law in Europe^16^ is to protect the fundamental rights of ‘data subjects’ – those whose personal data is processed by another person or entity – and to promote the smooth flow of personal data across systems and countries for economic and social benefit. It accomplishes this two-pronged function by making ‘personal data’ the object of the law. Behaviours of key actors – specifically ‘data controllers’ and ‘data processors’ – are steered through rules governing how they may use the personal data of data subjects. Once the actions of the persons in question generate, process, or *in any way* interact with the category of ‘personal data’, they are immediately brought within the purview of data protection law and the said persons must discharge a range of associated responsibilities to ensure that their actions are lawful. Conversely, if those actors are not dealing with ‘personal data’, then their actions are not subject to this legal regime. Thus, much of the discussion in the field of data protection is concerned not only with *what* constitutes ‘personal data’ but also *when* does personal data exist. The obvious example is the technique of adequately anonymised data which renders it non-personal for the purposes of this law.

To take another example, the function of clinical trials law in Europe^17^ is to protect the rights, safety, dignity and well-being of research participants (research subjects) in ways that – at the same time – generate reliable and robust data and avoid unnecessary administrative delays for starting a clinical trial, thus making Europe a relatively attractive place to conduct research. Here, desirable human behaviour of key actors – specifically clinical trial sponsors and investigators – is steered through rules governing how they may set up and run a clinical trial. The object of the law is the ‘clinical trial’, specifically to test medicinal products for human use. Both data protection law and clinical trials law fall within the larger category of the law governing scientific research involving humans, though of course each of these laws may fall also within other larger categories (e.g. the law governing commercial entities, the law governing the marketing of pharmaceuticals).

While we might accept *prima facie* that law and regulation need to ‘object-ify’ and categorise to fulfil their core functions, in the context of health research, we must also recognise that this can yield problematic outcomes in certain areas of law. Through its object categorisation, the law can inadvertently spur an overly technocratic view of the world that pays insufficient attention to human subjectivity and experience. Arguably, this is most pronounced in the health research sphere precisely because the human serves here as *both* subject and object. Because the human is at the centre of health research – as an investigator, participant, and ultimate beneficiary of the research – it is not enough for law and regulation merely to steer the behaviour of those who interact directly with those human ‘subjects’ to do so in ethically and socially desirable ways (whatever that might entail). That is, the focus of law ought not to be simply at the locus of the researcher-participant interaction, e.g. when recruiting someone to a clinical trial; nor should it only be at the interface of clinician/researcher and her patient/research participant; nor only on the biobanker and the responsible management of her cohort of participants. This is not to deny that these are crucially important foci of legal attention: the inter-subjective relationships involved are vital to the entire research enterprise, and they are founded on the human value of trust. But, it is precisely the entire research enterprise that is at stake and that should be under scrutiny. Trust must be maintained throughout the totality of the research process, i.e. from design and approval of the research protocol to the realisation and delivery of social value from the research itself. Yet, from a legal standpoint, as the research participant becomes less physically proximate to the research – that is, as the human subject becomes reduced to research ‘objects’ such as ‘personal data’ or ‘human tissue’ – the focus of law shifts, and some important human interests are overlooked as a result.

Human ‘subjects’ all too easily become the diced and deconstructed ‘objects’ of the law by having their component parts stripped down and gridded within artificial boundaries and categories that do not necessarily reflect the expectations or experiences of participants who take part in research. Personal data, embryos, cells, DNA, blood, and organs are both physically and legally excised from the body and slotted within silos or sections of rules. This observation in no way denies that protections do occur across this process, but they appear in isolation and at disparate junctures under diverse legal regimes. For example, there are no legal mechanisms to trace how far and how well research participants’ interests are protected across the entire research trajectory. The ‘promise’ of research made to the research ethics committee (which, we note, is before the research even begins) is not always adequately monitored over time;^18^ trust remains fragile throughout these processes, and can be broken for want of a clear social licence;^19^ downstream uses of research data and materials remain a legitimate concern of citizens, notably commercial access and use, even when core privacy and physical interests have been well-protected.^20^ All of this suggests that the siloed approach of law provides an incomplete picture, at least from the perspective of the human experience of being involved in research.

How then are we to make sense of this? We do not challenge the need for law and regulation to categorise, but we do suggest that in the process of object categorisation in health research, law and regulation could do better to (re)incorporate the subject (the participant) into the processes of human HRR. In other words, by better understanding how law and regulation create objects and categories of objects and, in so doing, separate the human subject from object, we can consider the ways in which we may devise legal and regulatory frameworks that better account for any enduring connections between subjects and objects. This analysis, in turn, can help to minimise disruptions or controversies that can arise in health research. To do so, we now turn to liminality, an anthropological concept concerned with processes of transformation and change.

## 3. Transitions between subjects and objects in health research: three case studies

In case study one, *From Object to Subject*, we argue that important normative work is done by recognising the identity interests that citizens have and retain throughout the research endeavour, that is, by recognising that research practices and findings can have important impacts on participants’ own identities to the extent that the experience of participation contributes to or detracts from their own narratives about who they are as persons.

In case study two, *From Subject to Object*, we suggest that governance frameworks that over-emphasise mechanisms such as the anonymisation of research participants’ data, or the role of informed consent, fail adequately to account for the interests that individuals might retain in their data, even when this may be no longer identifiable or ‘connected’ in the eyes of the law.

Finally, in case study three, *Between Subject and Object*, we offer insights into how an inherently liminal being – the human embryo – is currently categorised. We argue that the failure to recognise what it means to lead embryos out of liminality towards diverse ends of either reproduction or research use, make current regulations increasingly morally questionable. Moreover, we suggest that the liminal analysis offered here coupled with the normative account of what it means to be a research participant, viz. the experiences of women and men as progenitors and donors, can provide good reasons why gamete and embryo donors ought to have more of a say in the research that is done with and on their embryos.

### 3.1. From object to subject: constituting the identity of the research participant

In ordinary language, there is ample room for ambiguity about whether participants in health research may most appropriately be thought of as the *objects* or the *subjects* of the endeavour. In one sense, they may be seen as the objects of study – passive parties to whom research activities are done.^21^ This is perhaps most marked where research is conducted using data such as patient records, or stored tissue samples, without any direct contact with, or active involvement of, the participant. However, regulatory instruments and ethics guidelines governing health research commonly refer to ‘research subjects’ or ‘trial subjects’.^22^ This is fitting inasmuch as participants’ bodies, behaviours, tissues or data are the focus and material – the subject matter – of inquiry. Nevertheless, it is not always or obviously the case that the participant – qua *person* – is the true subject of research, when most studies aim not to investigate individual-level traits or health, but to develop generalisable knowledge derived from analysis of a large amount of aggregate information on multiple participants.

This section will propose one important reason why we should not lose sight of the research participant *as subject* in one important sense, even if we do not always use this term to refer to them. This is because the language of subjecthood serves to highlight an ethically significant aspect of the process and experience of participation.^23^ That is, taking part in health research can serve to construct the participant as the subject of her own life and experiences, by contributing to the development of her identity in a more thoroughgoing and normatively significant way than by merely acquiring the label of ‘research participant’.

#### 3.1.1. Narrative self-constitution

To make sense of these suggestions, it is necessary to say a little more about what it might mean for someone to have and to develop an identity. Although the account outlined here is only one possible way of conceptualising what makes each of us the particular individuals we are, it is one that resonates with many of our everyday ideas about what identity looks like and how it works; it is also receiving increasing (though not universal) support in philosophical and bioethics debates.^24^

A narrative conception of identity holds that each of our identities is constituted by the story we would each give of who we are. This story includes, amongst other features, our accounts of what we have experienced, what we have done and plan to do, our characteristics, desires, beliefs and values, and our relationships with other people, our bodies and the world around us. It is thus a constellation of many kinds of experiences, self-descriptions and modes of self-understanding. And it is a ‘story’, not only in the sense that extends and develops over time, but also because the features of which it comprises are not discrete elements, but part of a whole from which these various features derive their role, meaning and significance. A key feature of narrative theories of identity is the claim that our self-narratives do not merely describe us, but actually constitute who we are. An individual is the subject of her own self-narrative, in the sense that she is the protagonist, albeit one whose story is shaped by others and her environment, including that of her own body.^25^ And the contents and nature of her self-narrative provide the interpretive and evaluative frame through which she experiences herself and the world, it shapes her particular subjectivity.

#### 3.1.2. The impacts of participation

We will first examine the suggestion that the sheer act of taking part in research may itself contribute to the constitution of the participant as a subject. The proposal here is that taking part could provide experiences or plotlines that feed into an individual’s identity narrative and present opportunities for particular characteristics or self-descriptors to come to the fore or recede within this narrative.

According to a narrative conception of identity, our self-narratives are interpretive and ‘curated’ stories, in which some aspects of our lives feature (some prominently, others less so), while others do not.^26^ At the most straightforward level, then, taking part in health research is a plausible candidate for acquiring a place in an individual’s account of who they are simply because, for most of us, it is not a quotidian activity. It will bring novel interactions and experiences, and often involve unusual exposure to risk or scrutiny and a degree of burden. It may well then stand out as noteworthy amongst life’s other activities and thus ‘make it into’ someone’s account of who she is.

Similarly, as one of us has argued elsewhere,^27^ to the extent that taking part in research entails encounters with information about one’s own health, body or relationships in the form of research findings (either aggregate results or individually-relevant observations),^28^ these too may impact upon participants’ accounts of who they are. They may do so, for example, by adding, subtracting, reinforcing or reconfiguring threads of participants’ self-characterisations, including their relationships to others. Such findings might include, for example, results indicating increased susceptibility to mental illness, or those that cast doubt on assumed genetic heritage.^29^ To be clear, the suggestion here is not that research findings reveal who a participant ‘really is’, but rather that they may affect the ways in which participants construct the stories that constitute their identities.

However, even allowing that research findings and other experiences associated with participation may feed into someone’s self-narrative, it might still be queried whether the addition of even novel and noteworthy life experiences or insights supplied by research findings actually makes any substantial difference to *who someone is,* to their own identity and subjectivity. One response to this is that these new narrative threads do not occupy an isolated role in our identity narratives. Rather they serve to contextualise, colour and reconfigure other aspects of these stories and add to the lens through which we view and interpret our other experiences. A further response is that the experience of participation, and perhaps particularly the *choice* to do so in the first place, may be seen as playing normatively, not merely qualitatively, significant roles in constituting the participant as someone with particular commitments and values. This suggestion needs to be unpacked a little further.

Empirical studies that have investigated people’s motivations for taking part in various kinds of health research suggest that there are a number of possible ways that participation could contribute to the construction of the participant as a ‘moral subject’. Beyond being motivated by potential clinical benefits (for example access to check-ups or therapeutic interventions, which some may hope to gain from taking part),^30^ it is not uncommon for individuals to characterise their participation as an expression of their principles, values and concern for others. For example, they may report being motivated by a desire to help others – either future patients with a particular condition or, more generally, by contributing to the generation of scientific knowledge as a public good.^31^ Conversely, they may be less willing to participate when they doubt the social value of a study. Particularly in the field of genetic research – due to the inherently shared nature of genetic data and family histories – taking part may be a manifestation of particularly relational aspects of participants’ identities and associated bonds of care and concern for others. For example, participants undergoing testing to improve understanding of genetic diseases that run in their families may express their motives in terms of concern and love for relatives who could be at risk, or be a means of honouring those lost to the disease.^32^ Meanwhile, a quite distinct way in which participation may function as a kind of enactment of self-image, may be witnessed amongst participants who report that donating their genomic data to research via commercial direct-to-consumer services is a way of manifesting their identities as techno-pioneers or ‘early adopters’.^33^

The proposal here is that participation may do more than simply *express* some of an individual’s identifying characteristics. If we accept that to participate in health research is to enter into a liminal transformative process, then the introduction of the analytical frame of narrative identity offers a stronger interpretation – that taking part can actually serve to *constitute* who someone is. This is grounded in the claim that a self-narrative is not simply a set of inert self-descriptors, adopted or rejected at will. Rather, the relationship between one’s self-narrative and conduct is practical, normative and reflexive, meaning that the self-descriptors we adopt only plausibly define who we are to the extent that we actually act (or choose or judge) in accordance with them when it is possible and appropriate to do so.^34^ According to this analysis, then, participation is constitutive of identity to the extent that it provides opportunities for important threads of self-characterisation to be enacted and thus reinforced as part of the kind of person someone is. This offers a way of interpreting the inferences that Hallowell and colleagues draw from their own observations – that participants in health research ‘construct themselves as responsible and caring individuals’ and as ‘moral being[s]’ – in such a way that ‘construct’ can be understood literally, not as merely in terms of self-presentation.^35^ On this view, taking part in a genetic study (for example) may not merely demonstrate someone’s affection and concern for her (potentially at risk) siblings and children, but actually comprise part of what it means to her to *be* a loving parent and sister.^36^

According to the same reasoning, if it is plausible that acting in accordance with the characteristics with which we define ourselves reinforces their roles in our identities, then the converse will also hold. That is, taking part in research that is at odds with our values or other characteristics with which we identify – for example, as might be the case if it serves commercial interests rather than the interests of patients, or uses unsafe or unethical methods^37^ – may undermine, and be experienced as undermining, the corresponding aspects of our self-narratives and thus threaten our existing, and valued, accounts of who we are. Where the aims or conduct of studies are antithetical to the self-conception of participants, or the experience of participation is in some way distressing or contrary to their expectations, this could instead disrupt, or re-configure their identities in ways that are unwelcome or that that they struggle to inhabit or make sense of.

The suggestions made here, then, are that taking part in health research can be (re)constitutive of the participant as subject: the subject of her own identity narrative and her perspective on the world. As a result of participation, the particular self-descriptors, roles, relationships and experiences that make up an individual’s self-conception and provides the qualities of her subjectivity, may be gained, lost or rearranged in ways that are more, or less, welcome, desirable or ‘inhabitable’. Of course, the participant exists as a subject prior to participation but, through consenting and committing to participate and permitting herself to be the object of scrutiny and inquiry, she continues on the perpetual journey of evolving and becoming the particular subject she is.

Participation in health research may therefore be seen as a *process* through which, in serving as the object of research, the participant undergoes and experiences development as a subject, for better or perhaps worse. This transition from being an object of study to a particular subject is even more apparent where the means of participation is via literal objects – that is where it does not require the involvement of, or interventions upon, the whole person, but rather research conducted upon their data or tissue samples in their absence. As described above, the health research landscape is changing. Increasingly it is characterised by secondary data uses and studies that apply AI and bioinformatics to vast datasets.^38^ ‘Remote’ participation of the kind entailed by these types of studies might not entail the kind of notable or risky life experiences described earlier in this section, and thus not feature as narrative plotlines on these grounds. However, it may still result in the generation of personally significant research findings. And, as noted above, many of the empirical studies investigating motivations for participation focus precisely on the role of data-focused research in the construction of the ‘moral subject’. These studies provide some indication that this aspect of identity constitution is present even in data-led non-contact research. In this, we may witness the very epitome of the transition from research object to research subject: the constitution of the participant as subject from the objects of study, for example, her health records or tissue samples.

#### 3.1.3. Implications for managing health research

It might be queried why the potential impacts of health participation on our identities would, or should, make any difference to how health research is regulated. After all, if each of our identities is perpetually in development, why worry about one possible route of development? However, this is to overlook the implicit normativity in narrative conceptions of identity, normativity that is indicated in the examples given above. That is, we care about what kinds of individuals we are. We have interests in being able to develop and maintain aspects of our identities, and in being able to make sense of and comfortably inhabit our own accounts of who we are.^39^ If this is indeed the case, it carries ethical implications for practices that impinge on these capacities, amongst which – we suggest here – health research practices may be counted. This, in turn, has implications for the responsibilities of those conducting health research and the policies governing its ethical conduct. Seen through our liminal lens, the process of becoming and being a research participant makes it incumbent on those responsible for research processes to lead participants through and out of the entire research trajectory.

What, then, are some of the ways in which current health research regulatory norms and practices might recognise and respond to the ways that participation may serve to constitute the participant as subject? Here, we make only brief suggestions that will be picked up further in the discussion of a processual approach to regulation in Part 5. First, this analysis emphasises that the value of trust in the research relationship – and efforts to foster and protect this – are not only important for reasons of recruitment and social licence.^40^ In particular, it underscores the need to characterise fully and accurately the nature and aims of a study in recruiting participants and obtaining their consent to participate so that they can properly appraise how it fits with their ideas of who they are and their life projects. Also implicated are policies regarding the return of individually relevant research findings to participants.^41^ The preceding analysis suggests a need to recognise the potential significance of research findings to participants beyond their clinical actionability.^42^

Lastly, a key implication of this first example of the relationship between object and subject in health research is that the need to attend to possible impacts on participants’ identities are not severed by their physical remoteness from the research that is conducted, but may be mediated by their donation of research objects such as data or tissues. In the next example, we take a closer look at this relationship in the context of data governance, specifically the consent-or-anonymise binary, which is paradigmatic of the way in which the subject-object distinction draws focus away from the transformative experiences of the research participant.

### 3.2. From subject to object: the anonymisation of data

Big data provides us with an illustration of the problems associated with attempting to categorise and distinguish subjects and objects as separate, fixed constructs, as well as the consequences of prioritising the object over the subject. Here, we use the term ‘subject’ to refer to individuals to whom data pertains. We use the term ‘object’ to refer to the data and information which relate to the data subject. In this section, we consider the problems associated with current approaches to conceptualising subject/object relationships in the data context. In particular, we suggest that the dominant ‘consent-or-anonymise’ model represents a caricature of subject/object separations created, to some extent, by current regulatory frameworks and the ways in which big data strives to prioritise the object at the expense of important considerations around the subject, including narrative interests as outlined above. In this realm, we find that the law seeks to impose bright lines/thresholds across the data reuse research endeavour, and incorrectly assumes that these are reflective of the realities, including human experiences around data use.

However, we also acknowledge that research practice has a part to play here. A liminal approach, which emphasises the human experience and the process of transformation involved in health research, requires us to revisit the current constructs provided for within the law and to account for the subjects’ interests in their data and connections to them. Taken together, we suggest that governance frameworks that over-emphasise mechanisms, such as the anonymisation of research participants’ data, or the role of informed consent, fail to adequately account for the interests that individuals might retain in their data, even when this may be no longer identifiable or ‘connected’ in the eyes of the law. Data protection law makes the privacy-related interest of ‘identifiability’ the core concern; however, in addition to narrative identity interests, research subjects might also have reputation-related interests at stake – for example, not being associated with, or facilitating, research with which they fundamentally disapprove on moral grounds.

Alongside the clear benefits of data use and reuse, not least the rich insights these may provide across a wide variety of health, health-related, and even non-health concerns, come significant regulatory challenges.^43^ These include important questions around consent, privacy and the role and value of public interest, which we have discussed at length elsewhere.^44^ What has not received attention to date, but which is central to how we approach the regulation of data or research, are the ways in which the big data revolution, powered in part by secondary uses of patient and research data and the analytical capacities of artificial intelligence, is shifting the focus away from data subjects and towards big data objects. We see this reflected perhaps most starkly in the ‘consent-or-anonymise’ paradigm that, in our view, presents a false choice to data subjects as to the (ongoing) connections they may have with their data; it also fails to recognise the full range of subjects’ interests that are at stake.

#### 3.2.1. The consent-or-anonymise paradigm

As mentioned above, law’s creation of regulatory silos that attempt to draw bright lines around distinct categories of data (identifiable, anonymous, sensitive) is based on an assumption that such bright lines necessarily capture and sufficiently protect the core interests at stake. Indeed, in the case of anonymisation, the working assumption is that these technical processes adequately sever the relationships between data subjects and data objects: by rendering the data no longer ‘personal’, it also can no longer attach to a data subject. Under data protection law, anonymous data means ‘information which does not relate to an identified or identifiable natural person or to personal data rendered anonymous in such a manner that the data subject is not or no longer identifiable’.^45^ We suggest that such an assumption of relationship-severing is problematic in that it overlooks the important on-going (and at times implicit or symbolic) relationships between subjects and objects in terms of their interests in how data pertaining to them may be used including: who data are shared with (e.g. public/private organisations?), the purposes for data sharing (what kinds of research?) and the outcomes (does data use lead to commercial profit? If so, how are these profits used?).

The law’s relatively myopic approach results in prioritising legal focus on the object, i.e. the data relating to the individual. This is particularly so when data objects are used in large-scale datasets where the emphasis is placed on maximising the potential research value of the data objects. As datasets increase in volume and are continually linked to additional datasets, not only are the risks to privacy increased, but the opportunities to (re)create the legal category of ‘personal data’ are multiplied (because the likelihood of re-identification of individuals can increase through data linkage). This means, in practice, multiple third parties within a big data environment might become data controllers for the purposes of data protection law, so coming within this legal domain. Moreover, from the data subject’s perspective, as her data move further and further away from her immediate control and knowledge of what is being done, her influence is weakened or removed altogether while her interests in her data in terms of how it may be used, with whom it may be shared, and so on, remain the same – and become arguably stronger in some cases, e.g. if non-approved uses are made that impact negatively on reputational or identity interests.

Taken together, these examples show why the ‘consent-or-anonymise’ paradigm is increasingly problematic, in that it does not adequately reflect the interests that may be retained in how data is used, even if an individual is not identifiable in a legal sense. This is not to say that such interests should always be determinative – such an approach would be disproportionately detrimental to health research – but, as we will go on to argue below, neither should they be wholly discounted or ignored.

Consider, for example, the use of personally identifiable information versus non-identifiable data, and the distinct regulatory frameworks which each triggers. Personally identifiable information is regarded as information which relates to an identified or identifiable individual. Subject to certain caveats, to use personally identifiable information, there are both legal and ethical requirements to obtain the consent of the data subject prior to use. This can be problematic. For example, obtaining consent is timely and cost-intensive, particularly considering that big data studies rely upon data relating to thousands of individuals. Often, research projects have time-limited funding which severely impedes the ability to contact every single individual to obtain their consent. Likewise, questions arise as to whether or not obtaining individual consent is always desirable when data subjects may not necessarily wish to be contacted to obtain permission for every single use of their data in research.

In recognition of the impracticalities (and impossibilities) of obtaining consent, regulatory responses have been developed to facilitate the use of data without consent requirements via (amongst other approaches) the use of anonymisation. Anonymisation involves techniques that make the identification of a data subject highly unlikely, and thus the use of such data no longer falls within legal requirements to obtain the data subject’s consent or another lawful basis before use. We add a caveat to this point by acknowledging that data protection law (at least in Europe) generally offers a variety of lawful bases to process personal data, of which consent is but one. In principle, then, the choice to researchers (as data controllers) is more akin to ‘anonymise the data or choose a lawful basis’; in health research, there are projects that tend not to operate on consent for the lawful basis to process personal data, such as retrospective chart reviews and epidemiological research.^46^ This said, it is through long-standing practice in health research (foremost through the normative weight placed on consent) that the lawful basis chosen by many researchers to process personal data in their projects is, indeed, consent. The result has been the perpetuation of the ‘consent-or-anonymise’ paradigm,^47^ where researchers wishing to use data either obtain consent from data subjects *or* anonymise the data prior to use.

#### 3.2.2. Implications for health research

This model can be viewed as a paradigmatic example of regulatory attempts to separate the subject (the person) from the object (their data); it suggests that merely through ‘stripping’ identifiable information from data pertaining to a subject, the connection between the data subject and object is severed. The practical, ethical and social realities are, however, far more complicated. Each time datasets are linked together, more and more information pertaining to an individual is connected and the potential for re-identification increases. Technological developments in data use have also provided increased means of generating data that is re-identifying of persons; this, in turn, renders ‘true anonymisation’ highly unlikely.^48^ Moreover, despite the benefits for a research agenda of obviating consent requirements, the use of anonymous data (where re-identification is no longer possible) is not always ideal in the research setting when both (i) identifiable information and (ii) the ability to link together multiple datasets (which cannot be done via anonymisation) provide richer datasets with greater research potential. Consent-or-anonymise threatens to thwart many big data objectives while at the same time creating illusions of control and protection that – in many instances – simply do not respect the moral connection between a subject and her data.

One technocratic solution which has been developed to mitigate the limitations of the consent or anonymise paradigm is pseudonymisation. This is an alternative methodology that provides a means of retaining the ability to link together data about individuals across multiple datasets, without re-identification. Identifiers within datasets are attributed codes/pseudonyms which are held separately from original datasets. Thus, the potential to re-identify individuals and link together multiple datasets is maintained, whilst also paying due regard to privacy concerns. Indeed, pseudonymisation demonstrates the fluid nature of data, from identifiable to non-identifiable and back to identifiable, which occupies the research space and which manifestly contrasts with the concept of separation between subject and object. Thus, the prominence of the data subject waxes and wanes over time, depending upon the research context. It could be argued that data objects in the case of pseudonymisation may simultaneously be considered to fall within both categories of identifiable and non-identifiable or, in fact, within neither category. This feature of in-betweenness could be seen as an example of the liminality of things – data themselves are in processes of transforming and becoming something else (notably, or possible future relevance to a data subject).^49^ As such, our liminal framing suggests, once again that there are continued obligations to lead the data objects – and the data subjects – through and out of this digital liminal phase, and potentially also to close feedback loops between research participants and research outcomes, as we have argued in detail elsewhere.^50^

We suggest that anonymisation does not absolve us of thinking about broader ethical issues and from considering the full range of interests that are engaged – be they collective (e.g. the benefits of research) or individual (e.g. narrative or reputational). This illustrates that our analysis is not merely descriptive, but also can also direct us towards governance frameworks that are able to speak to this nuanced relationship. Indeed, when we engage with the limitations of such an approach as a default – both in terms of the researchers’ needs for a rich dataset, as well as individual narrative and reputational interests in the use of the data, identifiable or not – this forces us to consider alternative approaches. We address what some of these might look like in Part 5.

By acknowledging the ebb and flow from subject to object, this also directs our attention to the potential move *between* object and subject. As we have seen, the legal and moral status of the entity as object or subject is critically ambiguous and depends on the vagaries of what is done to it and the regulatory paradigm under which this falls, as explored further in the following section where we turn to a case study that exemplifies this, embryos *in vitro*, which under UK law are attributed a ‘special status,’ not quite person (subject) or thing (object).

### 3.3. Between subject and object: the human embryo in vitro

The human embryo *in vitro* is paradigmatic of an entity that does not fit neatly into either of the legal categories of ‘subject’ or ‘object.’ Embryonic development is the most rapidly unfolding biological process in any stage of human life. This complexity is mirrored in legal frameworks that are, at the same time, detailed and ambiguous. This section explores that complexity and exemplifies the need not to dismiss categories completely; in some cases, it might be appropriate to make the implicit ‘object’ status of the embryo explicit. This analysis suggests that even where we recognise the need for categorisation in some cases, our normative claim regarding the importance of subjects’ experiences remains. In this case, there is room for the law to better account for the process and experience of *becoming* a donor, and their *experience* of their embryos becoming decidedly a research ‘object.’

#### 3.3.1. A ‘special’ status?

All *in vitro* embryos that are created, stored, used, implanted, and disposed of in the UK are governed by the Human Fertilisation and Embryology Act 1990 (as amended) (‘the 1990 Act’). The intellectual basis for this Act lies in the 1985 Warnock Report, which recommended that embryos created *in vitro* be afforded a ‘special status’. While the exact nature of this status was and remains unclear, we know that it involves affording embryos ‘respect’,^51^ and not treating them with ‘frivolity’. Every embryo created *in vitro* enjoys the Warnock Report’s ‘special status’; this is reflected in provisions for how embryos are to be used either in reproduction or in research; it is found in the rule against implanting human-animal hybrids;^52^ and it is the reason behind the 14-day time limit on research with human embryos.^53^ Like other frameworks for HRR (e.g. the Human Tissue Act 2004), the 1990 Act itself focuses primarily on embryos *in vitro* (for the purposes of this section, the ‘object’), and what we can and cannot do to them, while little recognition is given to the context from which the embryos are created or obtained.

But what category is assigned to the *in vitro* embryo by law: subject or object? It is arguable that embryos *in vitro* are treated neither as a legal subject nor as a legal object by the 1990 Act, but rather as something that falls *in between* this binary. The unarticulated construction of embryos in law as subject-objects has been a result of attempting to regulate an uncertain space and to accommodate potentially fundamentally conflicting values: that is, showing respect for the embryo while promoting reproductive medicine and embryonic research in the public interest, the last of which destroys the embryo in the process. And, as noted above, while all embryos created *in vitro* are governed by this ‘special status’, there are at least two distinct paths which they may go down once created (or unfrozen): (1) towards reproductive ends and (2) towards research ends. While it is clear, legally speaking, that embryos are neither subjects nor objects in the traditional sense – they do not have legal personhood, but neither are they a mere ‘thing’ and so cannot be property – their subject-ness or object-ness in research practice is arguably affected by the ultimate end for which they are used. As some of us have argued elsewhere:

The liminal states and the subject/object dyad are important for the future of artificial reproduction and embryo research because the notion of ‘the moral status of the embryo’ underpins the entire legal architecture of human reproductive regulation. A liminal perspective suggests, however, that at best, the law may be perpetuating a moral myth, and at worst, the compressed regulatory regime is fundamentally flawed.^54^

To expand on this further, if embryos are placed on a ‘reproductive path’, their treatment as a subject, rather than as an object, could be said to intensify. This is clearly not to say that they are immediately treated as subjects in law (e.g. with personhood), but more as a *subjects-to-be*. For example, clinics responsible for reproductive IVF must consider a host of factors when deciding whether to accept clients for treatment and many of these are about the well-being of the future person.^55^ Equally, once these embryos are implanted, the Abortion Act 1967 makes it increasingly difficult, as the foetus develops, for the pregnancy to be terminated. Conversely, placing embryos on a research path intensifies their treatment as ‘object’. Once determinedly a research embryo (whether created for research purposes, or donated), these entities may *only* be researched on and then disposed of; they can never cross to the reproductive path. Admittedly, there are strict limits on what one can and cannot do with them, and before 14 days (or before the primitive streak, whichever happens sooner), they must be disposed of. Thus, although they are ‘not nothing’^56^ in moral and legal terms, research embryos are, for all practical intents and purposes, treated as ‘artefacts’.^57^ In other words, they become legal objects. This does not mean that they cannot be ‘special’ in some continuing sense; many legal objects and ‘artefacts’ engender considerable amounts of legal protection that reflects the ways in which we ‘value’ them – for example, celebrated works of art. Thus, *object-hood* does not necessarily render the embryo as ‘nothing’, but at the end of the day, research embryos are still disposed of much like any other object that outlives its usefulness. Still, embryos can *matter* in other ways. For example, empirical research has shown that ‘Some participants understood existing trajectories (including embryo research) as feeding back into the field of reproduction, hence, maintaining narrative consistency of hope where fertility treatment provides a technical solution for childless people’, but it has also been shown that voices of donors have been ‘marginalised’ in the research process and debates about research.^58^ Therefore, not only is the process of becoming a donor ridden with anxiety,^59^ but so is the possibility of ‘their’ donated embryos contributing to the advancement of the very endeavour they are trying to achieve as fertility patients.

Our analysis thus far encourages us to embrace fluid, experiential aspects of being involved in health research, and this case study also provides the opportunity to call out certain processes for what they are. The reality is that there is no such thing as the unitary ‘*in vitro* embryo’ as a legal category. *In vitro* embryos are liminal entities when they are created, but the decision to place them on the reproductive or the research path has moral implications that must be recognised. In other words, there is an imperative, here, to make the implicit explicit: the embryo that becomes a research artefact is being treated as more object-like. The point in time when this occurs is a moral and ethical crossroads that should be acknowledged. Moreover, in doing so, we open the potential for a more honest debate about what we can and should do with the embryo as an artefact (object) – which is distinct to the embryo as a future person (subject). At the same time, the relationship that donors have (as subjects) with their embryos (as objects) is imbued with moral meaning that is not yet fully reflected in the legal and governance arrangements that are currently in place.

#### 3.3.2. Implications for health research

The processes of transformation and change in the regulation of embryos *in vitro* are radically different depending on the outcome that is envisioned for them, and the ultimate end points are diametrically in opposition: one results in life, the other in destruction. Van Gennep’s original formulation of liminality describes processes of change in time and space, but, as we have argued elsewhere, another way of thinking about liminality is to focus on the spatial and temporal aspects of the very processes under consideration. A liminal lens reveals that being in-between bounded legal categories is only ever a temporary state; indeed, liminality itself is often only a fleeting matter because the tendency is to proceed through a liminal state towards a transformative end point.^60^ For the *in vitro* embryo, these end points are practically and morally irreconcilable. Yet, it is arguable that the all-encompassing ‘special status’ is blind to this; conceptually, it severs *in vitro* embryos from the multiplicity of futures that the law has regulated for (i.e. reproductive use/implantation or research/disposal), and leaves out the possibility for constructive debates (inclusive of embryo/gamete donors) surrounding how any next steps in embryo research regulation should take place.^61^ It perpetuates a myth of considerably dubious moral character.

In sum, we suggest that not only are the categories of subject and object helpfully problematised by our liminal lens but also that the failure to create appropriate categories for those whose subject-ness or object-ness is temporally dependent undermines the ethical legitimacy of law in human health research. Recognising the research embryo as a legal object has several normative implications. For one, it can open up new avenues of research; it might also give reason to provide a framework that allows us to tell donors more accurately and clearly what will happen to their donated embryos, and to give them more input to those future research processes.

Ultimately, the analysis of these three case studies allows us to consider more deeply who has a say in research processes, depending on what these processes are. Indeed, if an option for further donor involvement in research were deemed desirable, it might enable us to address more fully the critique that donors can feel as if they are on the side-lines when it comes to research.^62^ As we have demonstrated in this section, the regulatory landscape in health research severs a moral, and personal, a connection that many research participants may have with their research contributions, be it tissue, data, or embryos. We, therefore, suggest the need to rethink regulation in processual terms, as an endeavour that occurs over a much longer time period than HRR currently recognises; health research is a liminal process, yet we fail to treat it as such.^63^ We consider how we might do so using our framework for processual regulation in the next section.

## 4. Beyond categorisation: a framework for processual regulation

### 4.1. Challenging the subject-object paradigm

Each of the three case studies above presents challenges to the suitability of a siloed approach to subjects and objects in HRR. Together, these challenges may be summarised as follows:

- The first case study, focusing on the subjectivity of the research participant, suggests that a powerful and important personal interest may be under-represented and under-recognised in HRR: the interest that research participants have in constructing and inhabiting their own identity narratives. This interest may be impacted, for better or worse by participants’ experiences of taking part in health research, and by the ways that HRR influences these experiences;
- The products of research, such as ‘research data’, can also feed into one’s identity. The second case study, the consent-or-anonymise paradigm in data governance, provides a concrete example of the ways in which the subject/object separation can prioritise the object (viz. data) over the identity or experiences of the subject; this can also have implications for a wider set of reputational interests;
- Embryos *in vitro* exemplify another research process that relates to legal subjects and objects that has, at least in part, been segmented by this enduring distinction. Not because it has been pushed neatly into either category, but because, here, we have not called out processes for what they are. Despite the ‘special status’ of embryos, their subject-ness or object-ness intensify as fertility or research processes go on, respectively; therefore
- While processes of transition and change happen to the participant and her contribution (e.g. data, embryos, or tissue), she (as a subject) and her interests in that contribution remain a constant in the research timeline. The full implications of this are not yet recognised in law.

Liminality, as a lens, has revealed several insights about the ways in which research occurs in practice, and the fluidity of research participants’ experiences (and relation to) their contributions. Liminality also focuses our attention on the subject as the experiencer, often going through a transformation of identity as the research processes take place, from being a participant to a person affected by research results. We have argued that a rigid separation of the categories of subject and object in HRR fails to reflect the reality of research practices, where, for example, tissue becomes data. Each of these bounded spaces relates to each other, but the nature of the frameworks surrounding them mean that the experiences of research participants as being either research subject and/or object, or their relation to their contributions (be it tissue, embryos, or otherwise), are insufficiently captured by current regulatory regimes. Our approach redirects regulatory attention from one chiefly focused upon legal ‘objects’ e.g. tissue, to one that highlights the experiences of human ‘subject,’ i.e. the research participant.

But how can law and regulation better reflect this changing materiality, not only in a physical sense but also in terms of their importance (i.e. how much they *matter*) to their subjects, i.e. to research participants? Moreover, how can law and regulation reflect the fluidity of connection between research participants and research objects, such as data, tissue and embryos? Each of the above case studies captures a process in health research: the process of becoming a research participant or ‘research subject’ and their experiences as their contributions (be these data, tissue, or embryos) become research objects. These processes, oftentimes fluid and unfixed,^64^ are a key feature of research practices that, as our case studies show, is not currently reflected by HRR. To better reflect the experiences of research participants in the regulation of health research, be it their connection to their contributions/objects (e.g. data), however great or small,^65^ we need a regulatory framework that accounts for the greyness of the spaces between research participants and research objects. This may mean we need to consider who has a say in research processes, when they get to have a say, and how they might do so. We argue that this may be done through a framework for ‘processual regulation’.

### 4.2. What is processual regulation?

As mentioned above, we have collectively and individually explored previously notions of processual regulation as it relates to HRR. Processual regulation is more than a ‘mere focus on process in regulation. […] ‘[S]uch an approach requires a temporal–spatial examination of regulatory spaces and practices as these are experienced by all actors, including the relationship of actors with the objects of regulation.’^66^ In a previous piece, we suggested that processual-oriented regulation has the following features:

- Over time, it recognises the flexibility and fluidity inherent in laboratory and clinical research;
- In space, it focuses on iterative interactions that adapt to new developments in science and medicine, as well as with changes in law and regulation; and
- Through experience, it reflects the complete investigative endeavour and is able, for example, to guide the different involved parties through the entire research process.^67^

One of us has built upon this first iteration in the context of the regulation of embryos *in vitro* to suggest a specific, ‘context-based approach’ which recognises (a) the multiplicity of intersecting pathways that the law leads embryos through (i.e. research, reproduction, PGD, freezing); (b) each of these pathways is relational i.e. dependent on the decisions and *experiences* of those who lead them *into, on* and *out* of those pathways; and (c) that at the end of those pathways there are only two possible outcomes: implantation in vivo, or destruction.^68^ This approach, borne from a *liminal lens*, brings together the experiential and time-sensitive aspects of the processes that *in vitro* embryos are taken through by donors, researchers, and other stakeholders. Another of us, in the context of public interest and the sharing of health research data, used a *processual lens* to suggest that ‘a fuller account of the public interest is provided through the application of a processual approach that pays attention to (1) a holistic view of the operation of law, beyond the statute book; (2) the dynamic nature both of law (broadly conceived) and publics’ views over time; and (3) the actors, activities and subjectivities that are in play’.^69^

### 4.3. A framework for processual regulation

In this article, we have used a liminal lens to establish that the experience and interests of research participants are a key normative basis for better recognising the continuing connection between subjects and objects in HRR. In this section, we put forward a new framework for processual regulation, building on our previous iterations (5.2, above) and our findings from our analysis of the above case studies. We argue that this framework helps us to consider broader governance tools, within and beyond HRR. By binding objects of health research, law overlooks the experiences of the research participants qua subject of research and the donors of data, tissue and embryos. We suggest an alternative regulatory perspective, *processual regulation*, which has the following key features:

1. Acknowledges and articulates the often-enduring connection between subjects and objects;
2. Reflects the reality that fluidity can occur between the categories of subject and object in health research;
3. Recognises the potential implications of these relationships for a subject/research participant’s identity; and
4. Embraces the view that health research needs to be seen holistically, as an endeavour that continues *beyond* the categorisation (subject, object, research, consent, etc.) that HRR currently acknowledges.

Our analysis therefore suggests a need for greater *transparency* of research processes and more commitment to opportunities for co-production of regulation. For example, this means that research participants should have the opportunity to have a greater knowledge of, if not a real say in, what happens to their research contributions. In practical terms, processual regulation may therefore mean giving subjects the following opportunities:

- Meaningful on-going engagement in and *after* the research processes for research participants;
- Input to research direction and access decisions on the use of research resources;
- Feedback, on a personal level, relating to the *use of* and *findings from* participant contributions to health research (including individually relevant, and ‘incidental’ or unexpected findings, not only aggregate results)^70^; and
- Opportunity to co-produce regulation with regulatory actors so that their experience and ongoing metaphysical connection to their research contribution, whatever form that may take – even when ‘disconnected’ from their body – is duly recognised and accounted for throughout the research lifecycle.

Our framework exposes the opportunity to introduce mechanisms that give participants the options to be fed back information about the uses to which their contributions are put and – where appropriate – to participate meaningfully in processes of deciding on the direction of research. Equally, we do not claim that research participants always have a marked interest in research objects deriving from them. Instead, it is the nuances of the fluidity that *can occur* between subject and objects that is inadequately reflected in HRR. More generally, it should be noted that we do not suggest that processual regulation is necessarily tied to liminality; this is simply offered as a lens that helps us more closely to interrogate the spaces *between* conventional legal spaces in which much of health research practice takes place. Moreover, we also do not suggest that processual regulation should be limited to the realm of health research. In today’s world, where lines between common legal categories are increasingly blurred (especially ‘person’ and ‘thing’), and a plethora of technological developments are taking place (much faster than law and regulation can keep up with), processual regulation as an adaptive framework can help us to tackle these relatively new challenges in law and society.

## 5. Conclusion

We began this article by highlighting law’s current approaches to categorisation; law almost always focuses on the ‘thing’ and not the person from which the thing derives. We explained that this is particularly acute in HRR, which takes its cue from law, where the subject/object divide is more pronounced and unstable as most ‘things’ in this field of regulation come from the human body. HRR has thus tended to adopt a siloed approach, severing research objects from research subjects. Our normative position, however, is that the connection between subject and object within and across regulatory environments in human health research is a matter of profound ethical and social importance, and the law must recognise and respond to this by capturing HRR holistically as a process over a much longer period of time than it currently acknowledges.

Liminality, an anthropological concept concerned with processes of transformation and change, enabled us to emphasise the enduring connectedness between subjects and objects in these contexts, specifically in three case studies: the identity of the research participant, the anonymisation of data, and the embryo *in vitro.* Through our analysis of these case studies, we showed that the relationship between subjects (oftentimes research participants) and their objects is not so easily severed.

Overall, our core contribution has been to suggest that the notion that material of enduring human value – such as personal data and tissue – can be stripped of its moral significance by spatio-temporal distance, or by techno-bureaucratic measures such as anonymisation, is simply inadequate as a grounding for ethically robust research regimes – that is, regimes which recognise and respect the interests engaged through experience of participating in health research. We therefore offered a framework for ‘processual regulation’ to better capture HRR as an experiential process of transformation and change, particularly as these impact on the interests of subjects/participants involved in the research. This framework has significant practical implications for research participants (subjects), whom we argue should be recognised as having an on-going relationship with their research objects throughout the research lifecycle.

## Footnotes

1 Graeme Laurie, ‘Liminality and the Limits of Law in Health Research Regulation: What Are We Missing in the Spaces In-Between?’ (2016) 25 *Medical Law Review* 47, 49.

2 Regulation (EU) 2016/679 of the European Parliament and of the Council of 27 April 2016 on the protection of natural persons with regard to the processing of personal data and on the free movement of such data, and repealing Directive 95/46/EC (General Data Protection Regulation) (herein ‘GDPR’).

3 Human Fertilisation and Embryology Act 1990 (as amended) s4A.

4 Samuel Taylor-Alexander and others, ‘Beyond Regulatory Compression: Confronting the Liminal Spaces of Health Research Regulation’ (2016) 8 *Law, Innovation and Technology* 149, 158.

5 *Moore v. Regents of the University of California*, 793 P.2d 479 (Cal. 1990).

6 *R v Department of Health, Ex Parte Source Informatics Ltd* [C.A. 2000] 1 All ER 786.

7 See GDPR (n 2) Recital 26, Data Protection Act 2018, Human Tissue Act 2004.

8 See, for example: Pam Carter, Graeme Laurie, and Mary Dixon-Woods, ‘The Social Licence for Research: Why Care. Data Ran into Trouble’ (2015) 41 *Journal of Medical Ethics* 404; Alexis Clarke, Annie Mitchell and Charles Abraham ‘Understanding Donation Experiences of Unspecified (Altruistic) Kidney Donors’ (2014) 19 *British Journal of Health Psychology* 393; Sarah Parry, ‘(Re) Constructing Embryos in Stem Cell Research: Exploring the Meaning of Embryos for People Involved in Fertility Treatments’ (2006) 62 *Social Science and Medicine* 2349.

9 Taylor-Alexander and others (n 4) 150.

10 See Taylor-Alexander and others (n 4); Laurie (n 1).

11 See Barney Glaser and Anselm Strauss, *Status Passage* (Routledge and Kegan Paul, 1971).

12 Ezekiel Emanuel, David Wendler and Christine Grady, ‘An Ethical Framework for Biomedical Ethics’ Ezekiel Emanuel and others (eds), *The Oxford Textbook of Clinical Research Ethics* (Oxford University Press, 2018) 127.

13 Agomoni Ganguli-Mitra, Edward Dove, Graeme Laurie, and Samuel Taylor-Alexander, ‘Reconfiguring Social Value in Health Research Through the Lens of Liminality’ (2017) 31 *Bioethics* 87, 89.

14 Taylor-Alexander and others (n 4); Catriona McMillan, *The Human Embryo In vitro: Breaking the Legal Stalemate* (CUP, forthcoming); Annie Sorbie, ‘Sharing Confidential Health Data for Research Purposes in the UK: Where are “Publics” in the Public Interest?’ (2019) 16 *Evidence and Policy* 249.

15 Austin Sarat and others, ‘The Concept of Boundaries in the Practices and Products of Sociolegal Scholarship: An Introduction’ in Austin Sarat and others (eds), *Crossing Boundaries: Traditions and Transformations in Law and Society Research* (Northwestern University Press, 1998) 3–4.

16 Specifically, as seen in the GDPR (n 2).

17 Specifically, as seen in the EU Clinical Trials Regulation 536/2014.

18 Edward Dove, *Regulatory of Stewardship of Health Research: Navigating Participant Protection and Research Promotion* (Edward Elgar, 2020).

19 Carter and others (n 8).

20 Sara Davidson and others, ‘Public Acceptability of Data Sharing Between the Public, Private and Third Sectors for Research Purposes’ (2013) Scottish Government; Ipsos Mori ‘The One-Way Mirror: Public Attitudes to Commercial Access to Health Data’ (2016) Report prepared for the Wellcome Trust.

21 Oonagh Corrigan, and Richard Tutton, ‘What’s in a Name? Subjects, Volunteers, Participants and Activists in Clinical Research’ (2006) 1 *Clinical Ethics* 101.

22 For example, the Declaration of Helsinki and The Medicines for Human Use (Clinical Trials) Regulations 2004. Though the language of ‘participant’, with its more positive connotations of inclusivity and respect, has become more prevalent over recent decades (see Corrigan and Tutton, n 21).

23 This is not necessarily to the exclusion of the terminology of ‘participant’. However, contrary to the idea that the term ‘subject’ is in some sense less respectful of the agency and individuality of the individual than the term ‘participant’, a possible implication of the proposal made here is that this terminology may actually serve to emphasise the impact of participation on these attributes.

24 See, for example, Kim Atkins and Catriona Mackenzie, *Practical Identity and Narrative Agency* (Routledge 2013), David DeGrazia, *Human Identity and Bioethics* (Cambridge University Press, 2005), Emily Postan, ‘Defining Ourselves: Personal Bioinformation as a Tool of Narrative Self-Conception’ (2016) 13 *Journal of Bioethical Inquiry* 1331; Marya Schechtman, *The Constitution of Selves* (Cornell University Press, 1996).

25 Postan (n 24).

26 Schechtman (n 24).

27 Postan (n 24).

28 That they will receive such findings is by no means a given, particularly when these are classed as ‘incidental’ to the core purpose of the research. See, for example, Susan Wolf and others, ‘Managing Incidental Findings in Human Subjects Research: Analysis and Recommendations’ (2008) 36 *Journal of Law, Medicine & Ethics* 219.

29 Postan (n 24); Kristof Van Assche, Serge Gutwirth, and Sigrid Sterckx, ‘Protecting Dignitary Interests of Biobank Research Participants: Lessons from *Havasupai Tribe v Arizona Board of Regents*’ (2013) 5 *Law, Innovation and Technology* 54.

30 It should not be assumed, however, that hopes for personal therapeutic benefit are necessarily distinct from identity development. It is possible that for some, participation offers a means of being informed and proactive in the face of risk or ill-health, and thus, embracing a responsible, engaged and biologised mode of self-identification, see Carlos Novas and Nikolas Rose, ‘Genetic Risk and the Birth of the Somatic Individual’ (2000) 29 *Economy and Society* 485.

31 Mary Dixon-Woods and Carolyn Tarrant, ‘Why Do People Cooperate with Medical Research? Findings from Three Studies’ (2009) 68 *Social Science & Medicine* 2215; Nina Hallowell and others, ‘An Investigation of Patients’ Motivations for Their Participation in Genetics-Related Research’ (2010) 36 *Journal of Medical Ethics* 37.

32 Hallowell and others (n 31); Ann Hurley and others, ‘Genetic Susceptibility for Alzheimer’s Disease: Why Did Adult Offspring Seek Testing?’ (2005) 20 *American Journal of Alzheimer’s Disease & Other Dementias* 374.

33 Richard Tutton and Barbara Prainsack, ‘Enterprising or Altruistic Selves? Making Up Research Subjects in Genetics Research’ (2011) 37 *Sociology of Health and Illness* 1081.

34 Christine Korsgaard, *Self-constitution: Agency, Identity, and Integrity* (Oxford University Press, 2009).

35 Hallowell and others (n 31) 43–44.

36 Lori d'Agincourt-Canning, ‘Genetic Testing for Hereditary Breast and Ovarian Cancer: Responsibility and Choice’ (2006) 16 *Qualitative Health Research* 97.

37 Dixon-Woods and Tarrant (n 31).

38 Graeme Laurie and Nayha Sethi, ‘Towards Principles–Based Approaches to Governance of Health–Related Research Using Personal Data’ (2013) 4 *European Journal of Risk Regulation* 43.

39 Postan (n 24).

40 Carter, Laurie and Dixon-Woods (n 8) 404–409.

41 Wolf and others (n 28).

42 Emily Postan, ‘Disclosure of Research Findings: Changing Roles and Relationships’ in Graeme Laurie and others (eds), *Cambridge Handbook of Health Research Regulation* (CUP, forthcoming).

43 Graeme Laurie and Nayha Sethi, ‘Information Governance of Use of Health-Related Data in Medical Research in Scotland: Current Practices and Future Scenarios’ (2011) University of Edinburgh School of Law Working Paper No. 2011/26.

44 Nayha Sethi and Graeme Laurie, ‘Delivering Proportionate Governance in the Era of eHealth: Making Linkage and Privacy Work Together’ (2013) 13 *Medical Law International* 168; Laurie and Sethi, ‘Towards Principles-based Approaches’ (n 38) 43–57; Sorbie (n 14).

45 GDPR (n 2), Recital 26.

46 Edward Dove and Jiahong Chen, ‘Should Consent for Data Processing Be Privileged in Health Research? A Comparative Legal Analysis’ (2020) 10 *International Data Privacy Law* 117.

47 Academy of Medical Sciences, *Personal Data for Public Good: Using Health Information in Medical Research* (AMC, 2006).

48 Paul Ohm, ‘Broken Promises of Privacy: Responding to the Surprising Failure of Anonymization’ (2010) 57 UCLA Law Review 1701.

49 Subjects may also retain interest in their data post-mortem. See Sethi and Laurie (n 51); Laurie and Sethi (n 45) 43–57; Sorbie (n 10).

50 Taylor-Alexander and others (n 4).

51 *Report of the Committee of Inquiry into Human Fertilisation and Embryology* (Cmnd 9314, 1984) (‘The Warnock Report’), 11.15.

52 Human Fertilisation and Embryology Act 1990 (as amended) s3(2), s3ZA.

53 Ibid s3(4).

54 Taylor-Alexander and others (n 4) 168.

55 E.g. the ‘welfare of the child principle’, contained in s13(5) of the Human Fertilisation and Embryology Act 1990 (as amended); and the age limit for IVF treatment, see National Institute for Health and Care Excellence, *Fertility Problems: Assessment and Treatment* (NICE, 2013).

56 Mary Ford, ‘Nothing and Not Nothing: Law’s Ambivalent Response to Transformation and Transgression at the Beginning of Life’ in Stephen Smith and Ronan Deazley (eds), *The Legal, Medical and Cultural Regulation of the Body: Transformation and Transgression* (Routledge, 2009).

57 John K Mason, ‘Discord and Disposal of Embryos’ (2004) 8 *Edinburgh Law Review* 84.

58 Parry (n 8) 2358.

59 Ibid.

60 We recognise that states of permanent liminality can exist. Indeed, the embryo that is frozen in perpetuity could be an example of this. Space does not permit us to discuss the implications of this particular scenario

61 Any amendment also needs robust, open enquiry into public attitudes, see Giulia Cavaliere, ‘A 14-Day Limit for Bioethics: The Debate Over Human Embryo Research’ (2017) 18 *BMC Medical Ethics* 38.

62 Parry (n 8).

63 Laurie (n 1) 47.

64 Although, as we have shown above in the third case study, *in vitro* embryos, sometimes these processes are indeed fixed.

65 See Parry (n 8).

66 Taylor-Alexander and others (n 4).

67 Ibid.

68 See McMillan (n 14).

69 Sorbie (n 14) 261.

70 It is recognised here that in the context of health research, which is increasingly exploratory, translational and reliant on algorithmic analysis of big data, a rigid distinction between intended and incidental research finding is increasingly unsupportable.

